# Genotypic variation in blueberry flower morphology and nectar reward content affects pollinator attraction in a diverse breeding population

**DOI:** 10.1186/s12870-024-05495-6

**Published:** 2024-08-29

**Authors:** Juliana Cromie, John J. Ternest, Andrew P. Komatz, Paul M. Adunola, Camila Azevedo, Rachel E. Mallinger, Patricio R. Muñoz

**Affiliations:** 1 Department of Horticultural Sciences, University of Florida, Gainesville, FL USA; 2 Department of Entomology and Nematology, University of Florida, Gainesville, FL USA; 3 Department of Statistics, Federal University of Viçosa, Viçosa, MG Brazil

**Keywords:** *Vaccinium*, Morphological traits, Floral rewards, Plant-pollinator interactions, Pollination, Heritability, Plant breeding

## Abstract

**Background:**

Pollination is crucial to obtaining optimal blueberry yield and fruit quality. Despite substantial investments in seasonal beekeeping services, blueberry producers consistently report suboptimal pollinator visitation and fruit set in some cultivars. Flower morphology and floral rewards are among the key factors that have shown to contribute to pollinator attraction, however little is known about their relative importance for improving yield in the context of plant breeding. Clarifying the relationships between flower morphology, nectar reward content, pollinator recruitment, and pollination outcomes, as well as their genetic components, can inform breeding priorities for enhancing blueberry production. In the present study, we measured ten flower and nectar traits and indices of successful pollination, including fruit set, seed count, and fruit weight in 38 southern highbush blueberry genotypes. Additionally, we assessed pollinator visitation frequency and foraging behavior over two growing seasons. Several statistical models were tested to optimize the prediction of pollinator visitation and pollination success, including partial least squares, BayesB, ridge-regression, and random forest.

**Results:**

Random forest models obtained high predictive abilities for pollinator visitation frequency, with values of 0.54, 0.52, and 0.66 for honey bee, bumble bee, and total pollinator visits, respectively. The BayesB model provided the most consistent prediction of fruit set, fruit weight, and seed set, with predictive abilities of 0.07, -0.08, and 0.42, respectively. Variable importance analysis revealed that genotypic differences in nectar volume had the greatest impact on honey bee and bumble bee visitation, although preferences for flower morphological traits varied depending on the foraging task. Flower density was a major driving factor attracting nectar-foraging honey bees and bumble bees, while pollen-foraging bumble bees were most influenced by flower accessibility, specifically corolla length and the length-to-width ratio.

**Conclusions:**

Honey bees comprised the majority of pollinator visits, and were primarily influenced by nectar volume and flower density. Corolla length and the length-to-width ratio were also identified as the main predictors of fruit set, fruit weight, seed count, as well as pollen-foraging bumble bee visits, suggesting that these bees and their foraging preferences may play a pivotal role in fruit production. Moderate to high narrow-sense heritability values (ranging from 0.30 to 0.77) were obtained for all floral traits, indicating that selective breeding efforts may enhance cultivar attractiveness to pollinators.

**Supplementary Information:**

The online version contains supplementary material available at 10.1186/s12870-024-05495-6.

## Background

Southern highbush blueberry (*Vaccinium corymbosum* hybrids) relies on cross-pollination for optimal production, with bees contributing the vast majority of pollination [1–4]. This places blueberry among the crops with the most demanding pollination requirements, as evidenced by substantial reductions in fruit set and berry quality in the absence of effective pollination [5–7]. To mitigate pollination-associated yield loss, blueberry producers in the United States spent more than $15 M on beekeeping services, or $363.63 per hectare ($147.20/acre) in 2022 [8]. Despite these substantial investments, blueberry still exhibits pollen limitation and subsequent yield loss [6, 9, 10]. As a result, recent reports have placed pollination and fruit set as a top priority for blueberry breeding, research, and development [11, 12].

While traditionally treated as a management concern, blueberry pollination has shown inconsistent results in response to increasing honey bee hive stocking densities [13–15]. Moreover, honey bees have proven to be suboptimal pollinators of blueberry flowers due to their inability to “buzz” pollinate, which is essential to shed pollen from blueberry’s poricidal anthers [2, 16, 17]. While the adoption of managed bumble bees is increasing due to their greater pollination efficacy, bumble bee stocking densities continue to lag behind those of honey bees in most regions, primarily due to their high purchase cost [1]. Consequently, efforts are underway to explore alternative solutions for improving pollination outcomes. Several studies have noted variability in pollinator attraction and fruit set among blueberry genotypes, prompting suggestions to breed pollinator-friendly cultivars with enhanced bee visitation, pollination efficacy, and crop yield [18, 19].

Flower morphology and nectar rewards are among the key genetically controlled factors that contribute to pollinator attraction and pollination efficacy [20–24]. Within this context, blueberry exhibits great variability for flower traits across species and genotypes [9, 25–27]. Characterized by a unique bell-shaped corolla, blueberry flowers secrete nectar from the base of the flower, which is made accessible through a narrow aperture at the distal end of the flower. This structure impedes nectar access for some pollinators, such as honey bees, which often leads to nectar robbery and subsequent pollen limitation [28]. A previous study comparing four highbush blueberry (*V. corymbosum* L.) cultivars found that cv. ‘Duke’, the cultivar displaying the highest rates of honey bee visitation and fruit set, also had significantly wider corollas and aperture diameters than other cultivars, signifying preferences for more accessible flowers [27]. An increased incidence of nectar robbery was also reported for the long and narrow flowers of cv. ‘Bluecrop’, which displayed significantly lower fruit set. Although distinct honey bee and bumble bee foraging patterns were noted between flower morphological traits, the limited phenotypic diversity observed did not allow for the resolution of trait preferences between pollinator species.

Morphological traits can also influence fruit set through the promotion of outcrossing, which is crucial for circumventing fruit abortion due to self-incompatibility or early-acting inbreeding depression [29–31]. Blueberry flowers exhibit herkogamy, wherein the stigma’s location within the corolla and the distance between the stigma and anthers reduce the incidence of self-pollination [32, 33]. In wild lowbush blueberry *(V. angustifolium)*, style length and the exertion of the stigma beyond the corolla were positively correlated with berry weight and the number of seeds per berry [34], suggesting these traits may be beneficial for pollinator attraction or pollination success. While significant variation for herkogamy has been observed in highbush blueberry [18, 27], its direct impact on seed and fruit set has yet to be examined.

Nectar volume and sugar concentration have shown to directly impact pollinator visitation in several crops, including blueberry, pepper, citrus, raspberry, blackberry, and sunflower [35–39]. Early investigations found that nectar sugar concentration of lowbush blueberry (*V. angustifolium* and *V. myrtilloides*) was correlated with higher fruit weight for one of two species, although results were not congruent between years [40]. Conversely, Jablonski et al. (1985) [35] reported a positive association between nectar volume, pollinator visitation rates, and fruit set, but found no significant relationship with nectar sugar concentration. Importantly, these authors also found substantial diversity in nectar volume and sugar concentration across fourteen highbush blueberry cultivars. Therefore, it is suspected that the content of floral rewards may contribute to genotypic differences in pollinator attraction and yield outcomes. However, preferences in nectar quantity and quality may vary between pollinator species. Additionally, the relative importance of nectar content with respect to other flower morphological traits has yet to be assessed.

Although several studies have found associations between nectar content and flower morphological traits with bee visitation or fruit set, current reports have focused on a small number of blueberry cultivars and wild species [25–27, 34, 40]. A comprehensive analysis that dissects the complex relationship between floral traits, bee behavior, and pollination outcomes is yet to be conducted for southern highbush blueberries. Therefore, in the present study, we assessed the relationship between ten flower morphological and nectar traits, pollinator visitation, and three indices of pollination success, including fruit set, seed count, and berry weight, across 38 southern highbush blueberry genotypes, replicated over two years. Specifically, we aimed to determine whether floral traits are heritable and quantifiably linked to genotypic variability. Additionally, we sought to clarify the role of these traits in pollinator attraction and pollination outcomes through various predictive analyses, and establish their relative importance to inform selective breeding efforts.

## Materials and methods

### Plant materials

This study included 38 southern highbush blueberry genotypes from the University of Florida Blueberry Breeding Program. These genotypes are part of a diverse breeding population and were selected for their variability in fruit setting and overlapping flowering time based on data collected in 2019–2020. Plants were managed in a two hectare trial plot on a commercial farm in Waldo, Florida, USA, considering an average spacing of 0.76 m within rows and 3.42 m between rows. Genotypes were intermixed within each row, and 15 clones were planted for each genotype. The genotypes included in this study were randomly distributed throughout the plot, which comprised a total of 330 genotypes. Data was collected over two years during the 2021 and 2022 seasons, when plants were six and seven years of age, respectively.

### Pollinator management

A total of 60 honey bee hives were stocked adjacent to the experimental plot to serve the entire farm, according to the farm’s management practices using approximately 10 hives/ha. Bumble bee quads, comprising four colonies per quad, were also placed adjacent to the plot in both years, with stocking densities of 2 quads/ha in 2021, and 1 quad/ha in 2022. In addition to managed bees, native insect pollinators could also have access to the plot throughout the bloom period.

### Flower morphological and nectar traits

Eight flower morphological traits were assessed, including corolla length, width, and length-to-width ratio, flower size (volume of cylinder, *V* ~ πr^2^h), aperture diameter, style length, stigma protrusion from the corolla, and anther-stigma distance. During peak bloom, flower samples comprising ten recently opened flowers were collected from three random clones of each genotype, totaling 30 flowers per genotype each season. These flowers were cross-sectioned, imaged, and the traits measured via ImageJ FIJI software [41].

To evaluate nectar volume and sugar concentration, three randomly selected clones per genotype were covered with pollinator exclusion netting during the bud stage to prevent nectar consumption. During anthesis, 30 newly opened flowers were collected from each clone, and samples were promptly stored at 4 °C until they could be processed within 24 h in the laboratory. For each clone, the 30 flowers were pooled together, resulting in one composite sample per clone or three composite samples per genotype. Nectar volume was quantified using calibrated microcapillary pipettes (Drummond Scientific Co., Broomhall, Pennsylvania, USA), and nectar sugar concentration was measured using a hand-held refractometer adapted for small volumes (Bellingham and Stanley, College Station, Texas, USA).

### Pollinator visitation observations

Individual one-minute pollinator observations were conducted on an individual bush for each of the 38 genotypes to measure pollinator visitation and foraging habits. On each sampling date, bushes were randomly selected for observation of each genotype. When weather conditions allowed, these observations were performed twice daily, at least three times per week, throughout the flowering period. All observations occurred between February 27 - March 16, 2021, and February 14–22, 2022. Genotypes were observed between 15 and 25 times across the two years, for a total of 856 unique one-minute observations. Pollinator visitation was recorded as the number of legitimate flower visits for honey bees, bumble bees, and other flower visitors during each observation. Bumble bee species were classified together, since the majority of visits were made by *Bombus impatiens*, and the different species can be challenging to identify in field settings. Bumble bee foraging behaviors were also recorded, discerning whether an individual was actively foraging for pollen or nectar. Pollen foraging was noted if an individual was carrying pollen in their corbiculae or if they were seen buzz pollinating. Those individuals that did not meet either of these criteria were determined to be nectar foraging. This distinction was not made for honey bees, which were nearly always foraging for nectar, or for other pollinators, which were few. Additionally, ‘flower density’, defined as the average number of observable flowers on each bush from a single vantage point during pollinator sampling, was measured and included in the statistical models as a fixed effect.

### Fruit set and pollination indices

Various pollination indices, including seed count, fruit set, and fruit weight, were measured for three clones of each genotype. The proportion of fruit set was determined by dividing the number of ripe fruits by the number of flowers for three open-pollinated branches per clone. Fruit weight was recorded as the mass of 25 ripe fruits per clone, harvested during the peak production window for each genotype, defined as when at least 70% of fruits for a genotype were ripe. After weighing, ten berries were randomly selected for seed count measurements. Seed count comprised the total number of filled seeds, defined as large, round, and dark in color.

### Phenotypic correlation and heritability estimation

To determine the relationships between floral traits and pollination indices, a Pearson’s correlation test was conducted. Estimations of heritability were carried out via a linear mixed model, expressed in matrix notation as: $\:y=1{\mu\:}\:+X\beta\:+Zu+e\:$(1), where ***y*** is the vector of phenotypic records for a specific trait; $\:\varvec{\mu\:}$ is the overall mean; $\:\varvec{\beta\:}$ is the fixed effect of year, ***u*** is the random effect of genetic values with $u \sim MVN{\rm{(}}0,A\sigma _a^2{\rm{)}}$, where ***A*** is the numerator relationship matrix that describes the genetic relatedness between individuals based on pedigree and $\:{\sigma\:}_{a}^{2}$ is the additive genetic variance, and ***e*** is the residual vector with $e \sim MVN{\rm{(}}0,I\sigma _e^2{\rm{)}}$, where ***I*** is the identity matrix and $\:{\sigma\:}_{e}^{2}$ is the residual variance. The ***A*** matrix was constructed using the ‘AGHmatrix’ R package [42], and ***X*** and ***Z*** are the incidence matrices for the fixed and random effects, respectively. Variance components were estimated using restricted maximum likelihood (REML) methodology [43]. The genetic values were estimated using the Henderson’s equation through best-linear unbiased prediction (BLUP) [44]. The heritability estimate is given by $\:{h}^{2}=\:\frac{{\sigma\:}_{a}^{2}}{{\sigma\:}_{a}^{2}+{\sigma\:}_{e}^{2}}$ (2). Mixed model analyses were conducted using the ‘ASReml-R’ package [45].

### Prediction of fruit set using pollinator visits

A linear mixed model was used to evaluate the predictive ability of pollinator visitation rates for fruit set across genotypes. This model treated pollination visits as a fixed effect and included both a random intercept and a random slope for pollination visits, enabling the prediction of each genotype’s independent response to pollinator visitation. The random regression was conducted using the ‘GMMAT’ R package [46]. To ensure these data met the assumptions of the model, residuals were inspected for normality and homogeneity of variance.

### Prediction of pollinator visitation and pollination indices using flower traits

We tested four statistical and machine learning models, including partial least squares (PLS), ridge-regression (RR), BayesB, and random forest (RF), for their ability to predict pollinator visitation frequency and indices of pollination success based on flower traits. Predictive abilities were assessed through a k-fold cross-validation process, using training set (80%) to test set (20%) ratio repeated 100 times. For statistical methods, we employed the linear model $\:\varvec{y}\:=\:1{\mu\:}\:+\mathbf{X}\varvec{\beta\:}+\mathbf{W}\mathbf{s}+\:\mathbf{e}$(3), where ***y*** is the vector of phenotypic records for a particular trait; ***μ*** is the overall mean; $\:\varvec{\beta\:}$ is the fixed effect of year with incidence matrix ***X***; ***W*** is the matrix containing the floral traits and flower density, ***s*** is the vector of their effects on the pollinator visitation and pollination indices, and ***e*** is the residual vector with $e \sim MVN{\rm{(}}0,I\sigma _e^2{\rm{)}}$, where ***I*** is the identity matrix and $\:{\sigma\:}_{e}^{2}$ is the residual variance.

Each statistical methodology comes with its own set of assumptions: PLS assumes that all effects within the model [2] are fixed effects and employs latent variables and the ordinary least squares method for estimating these effects. RR assumes ***s*** as random effects following a normal distribution with a common variance for all effects and utilizes REML-BLUP methodology for estimating these effects. BayesB is a Bayesian method and assumes ***s*** as random effects following a mixture of normal distribution with a proportion of effects that can be null effects, determined by their importance. As a representative of the machine learning approach, we used random forest (RF), a method based on tree algorithms that uses a third of the number of variables randomly sampled as candidates at each split [47]. The specific implementations include: PLS was fitted via the ‘pls’ R package [48], BayesB and RR were fitted via the ‘BGLR’ R package [49] and ‘rrBLUP’ R package [50], respectively. RF was fitted via the ‘randomForest’ R package [51]. In the Bayesian method, we used 300,000 total iterations for the Markov chain Monte Carlo algorithms and the first 20,000 iterations were discarded as burn-in. After every set of 5 iterations (thin), a sample was retained to calculate posterior estimates. The convergence of the Markov chains was verified through Geweke’s diagnostic [52].

### Variable importance analysis

Based on the results of previous prediction analyses, we selected the model that demonstrated superior performance in predicting pollinator visitation and pollination indices. Variable importance was determined by calculating the relative influence of each variable in relation to these specific pollination traits. For statistical methods, we considered the value of the normalized regression coefficient, while for the random forest method, we utilized a measure based on the usage of a specific variable at each split in each tree. The loss in the split-RSME (Root-Mean-Square Error) is the importance measure attributed to the splitting variable and is accumulated over all of the trees in the forest individually for each variable. To enable meaningful comparisons across traits, all variance importance values were standardized.

## Results

### Phenotypic diversity, correlation, and heritability

**Fig. 1:**
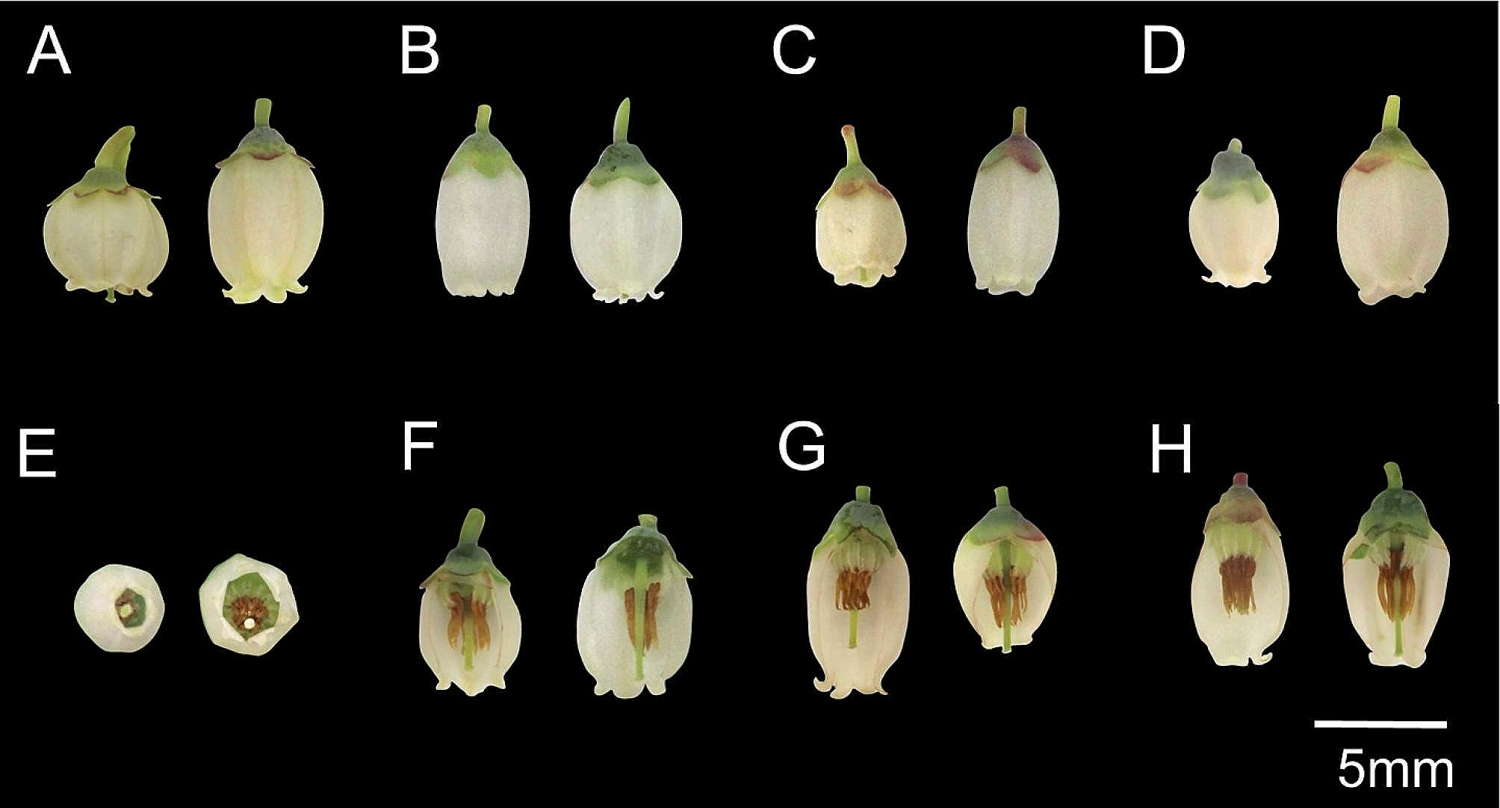
Floral diversity observed among 38 southern highbush blueberry genotypes for (**A**) corolla length, (**B**) corolla width, (**C**) ratio of corolla length-to-width, (**D**) flower size, (**E**) aperture diameter, (**F**) style length, (**G**) stigma protrusion, and (**H**) anther-to-stigma distance

Considerable phenotypic variability was observed for all flower traits across the 38 southern highbush blueberry genotypes (Fig. 1; Supplementary Figure S1).

**Fig. 2:**
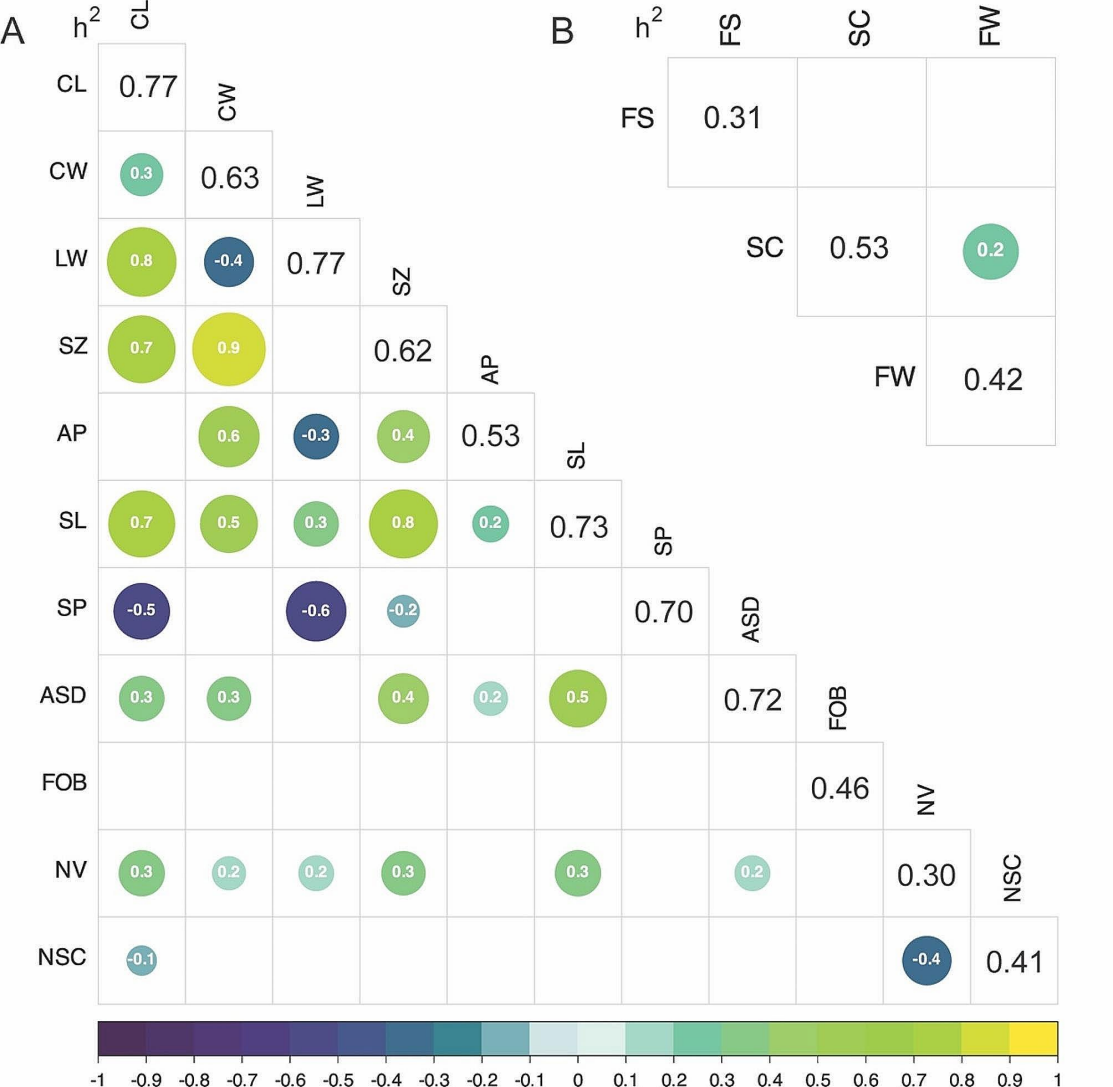
Narrow-sense heritability (diagonal) and Pearson’s correlation coefficients (off-diagonal) of (**A**) flower traits, and (**B**) pollination indices. The size and color of each dot is proportional to the magnitude and direction of the Pearson’s correlation coefficient, respectively. Only statistically significant relationships with *p* < 0.05 are displayed. CL, corolla length; CW, corolla width; LW, ratio of corolla length-to-width; SZ, flower size; AP, aperture diameter; SL, style length; SP, stigma protrusion from corolla; ASD, anther-to-stigma distance; FOB, flowers on bush; NV, nectar volume; NSC, nectar sugar concentration; FS, fruit set; SC, seed count; FW, fruit weight

A Pearson’s correlation test revealed significant associations between flower size and nearly all morphological traits, except for corolla aperture diameter and the corolla length-to-width ratio (*P* < 0.05; Fig. 2). Nectar volume and sugar concentration were negatively correlated (*r* = -0.38, *P* < 0.001), and the volume of nectar secreted was positively associated with flower size (*r* = 0.31, *P* < 0.001). A significant correlation between the pollination indices of seed count and fruit weight was also observed (*r* = 0.22, *P* < 0.01). The heritability values of all flower morphology and nectar traits were moderate to high, ranging from 0.30 to 0.77 (Fig. 2). Pollination indices showed moderate heritability values of 0.31, 0.53, and 0.42 for fruit set, seed count, and fruit weight, respectively.

### Genotypic differences in pollinator visitation

**Fig. 3:**
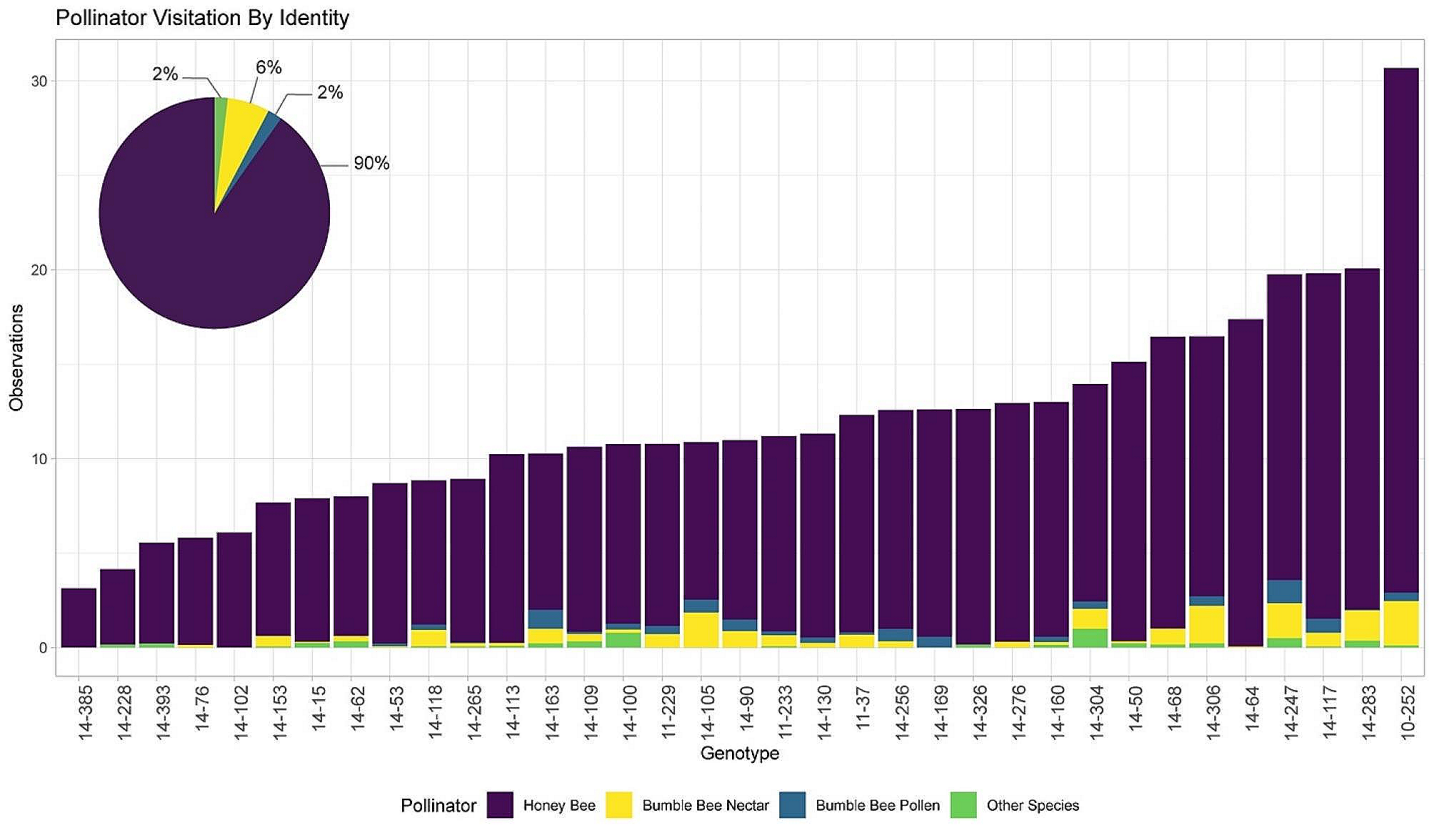
Total visits from each pollinator species (pie chart) and the total pollinator visits per genotype (bar plot), represented as the average number of pollinator visits observed per minute across both the 2021 and 2022 season. Colors indicate pollinator species and foraging behavior. Other species included carpenter bees, flower wasps, hover flies, and the southeastern blueberry bee

The total pollinator visits observed for each genotype ranged between 30 and 218 and 0 to 130 in the 2021 and 2022 seasons, respectively (Fig. 3; Supplementary Figure S2). In the 2022 season, there was a decrease in visitation rates across all genotypes. This decline could be due to the earlier bloom period observed in 2022 or variability in temperature and cloud cover, which are known to impact bee activity (Supplementary Figure S3). Most pollinator visits (90%) were made by honey bees (*Apis mellifera*), which exclusively foraged for nectar. Bumble bees, including *Bombus impatiens* and *Bombus bimaculatus*, comprised 8% of flower visits, of which 75% foraged for nectar, and the remaining 25% collected pollen. Other species comprised 2% of total flower visitors and consisted mostly of carpenter bees (*Xylocopa virginica* and *X. micans*), although a small number of visits were also made by southeastern blueberry bees (*Habropoda laboriosa*), flower wasps (*Scoliid spp*.), and hover flies (Syrphidae family).

In certain genotypes, honey bees comprised only 73% of all visits, while others were solely visited by honey bees. Additionally, we noted distinct preferences among bumble bees for specific genotypes, with their representation reaching up to 26% of all visits, while some genotypes received zero visits from bumble bees. Visits from other pollinator species were generally infrequent, and never exceeded 10% of the total pollinator visits for any genotype.

### The effect of pollinator visitation on fruit set was not significant across genotypes

**Fig. 4:**
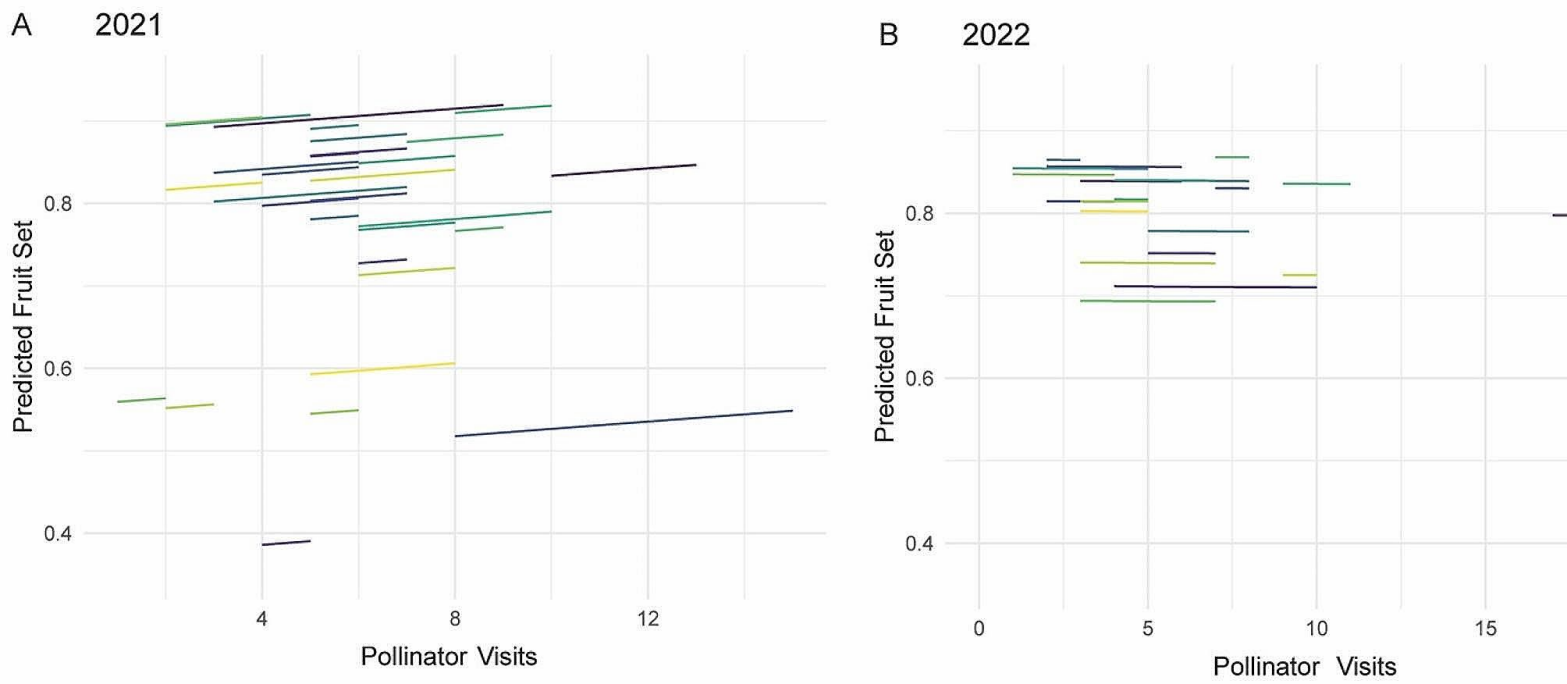
Fruit set predictions based on total pollination visits normalized by the number of flowers per bush during the 2021 and 2022 seasons. Each colored line represents a unique genotype

A linear mixed model was used to predict fruit set using pollinator visits in all 38 genotypes. However, the genetic effect was statistically non-significant when we evaluated the performance of the genotypes individually. Still, these genotypes exhibited some variability in fruit set regardless of visitation, suggesting that genotypic variability for fruit set may be mediated by factors other than pollinator visitation. Additionally, the relationship between pollinator visitation and fruit set varied between years (Fig. 4). In 2021, all genotypes displayed higher fruit set with increasing pollinator visitation frequency, but this pattern was not observed in 2022.

### Prediction models and variable importance analyses indicated floral trait preferences between bee species

**Fig. 5:**
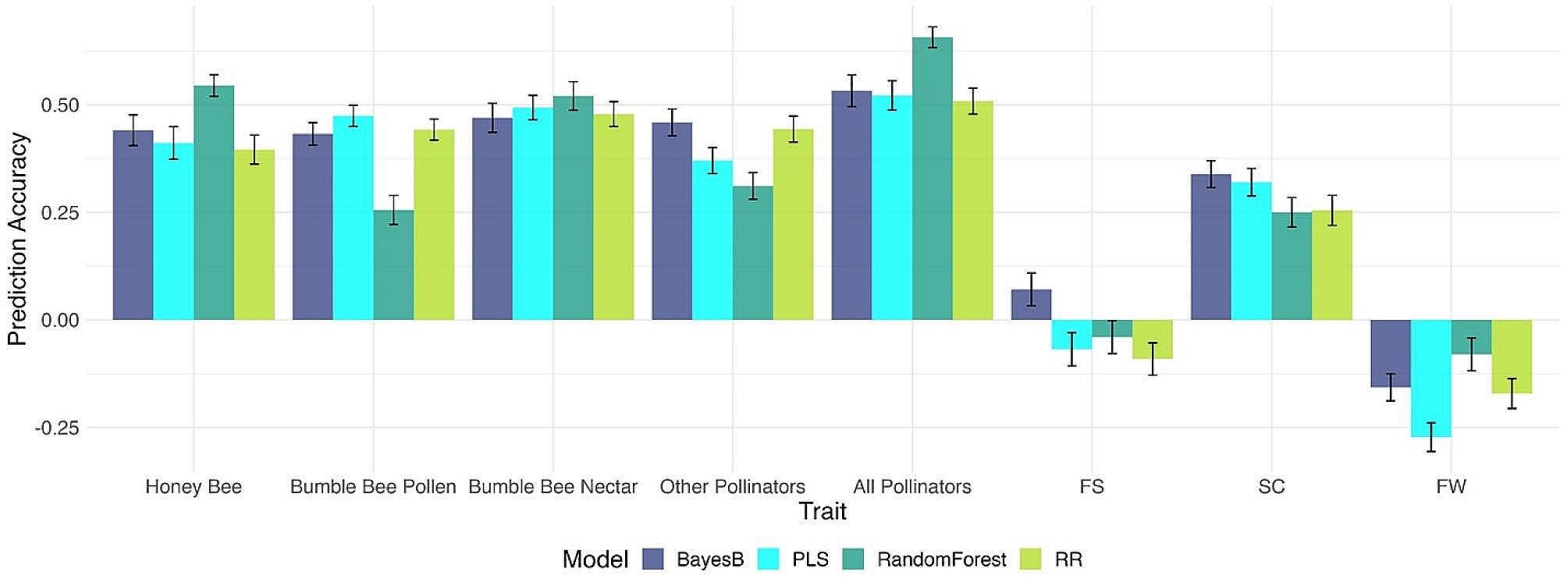
Predictive abilities for pollinator visitation and pollination indices using floral traits. Pollination indices include fruit set (FS), fruit weight (FW), and seed count (SC). Four prediction models were tested: BayesB, partial least squares (PLS), random forest, and ridge-regression (RR) models

PLS, BayesB, RR, and RF regression models were evaluated for their ability to predict pollinator visitation frequency and pollination indices using flower density, nectar rewards, and flower morphological traits as predictor variables (Fig. 5; Supplementary Figure S6). To determine if spatial variation contributed to pollinator visitation or pollination indices, the position of each genotype in the field, described as row and column were fitted as fixed effects. Neither row nor column were significant in both years, hence, they were excluded from the final model (Supplementary Figure S4). Additionally, three genotypes were removed from the analysis since they displayed higher than expected seed counts with low rates of pollinator visitation, and they also maintained a high degree of self-compatibility based on evaluation from another study (unpublished data; Supplementary Figure S5).

RF exhibited the highest predictive abilities for honey bee, nectar foraging bumble bee, and overall pollinator visitation frequency, with predictive abilities of 0.54, 0.52, and 0.66, respectively (Fig. 5). Therefore, RF models were employed to explore the relative contribution of each floral trait to the prediction of pollinator visitation in the variable importance analysis. Although the predictive abilities for pollination indices were generally low across all models, BayesB performed best, with values of 0.07, 0.34, and − 0.16 for fruit set, seed count, and fruit weight, respectively.

### The influence of floral traits on pollinator visitation rates depended on foraging task

**Fig. 6:**
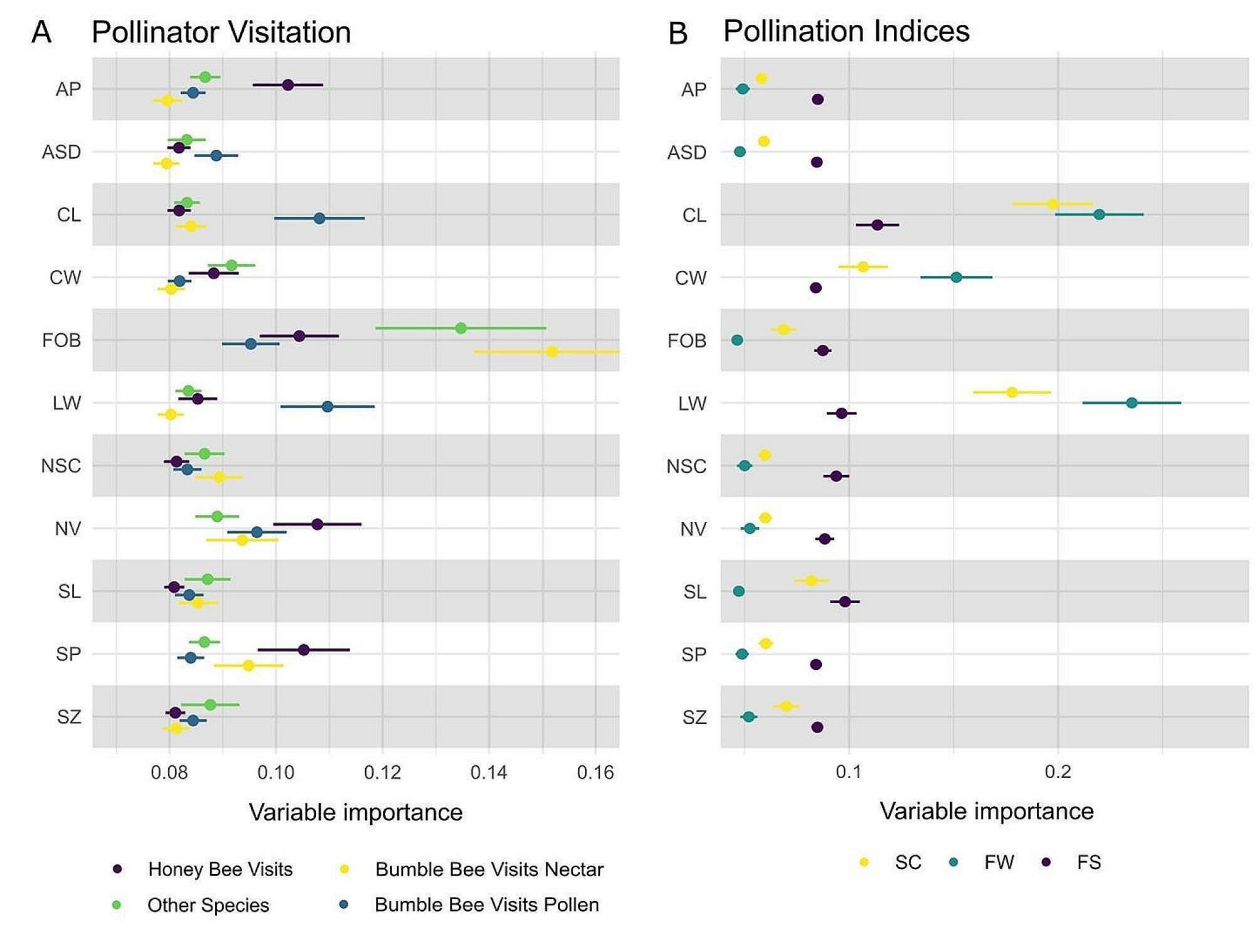
Variable importance shows the relevance of specific flower traits in predicting pollinator visitation (**A**) and pollination indices (**B**), using random forest and BayesB models, respectively. Higher values indicate greater importance. Flower traits: AP, aperture diameter; ASD, anther-to-stigma distance; CL, corolla length; CW, corolla width; FOB, flowers on bush (flowering density); LW, ratio of corolla length-to-width; NSC, nectar sugar content; NV, nectar volume; SL, style length; SP, stigma protrusion from corolla; SZ, flower size. Pollination indices: SC, seed count; FW, fruit weight; FS, fruit set

The relevance of each floral trait in the prediction of pollinator visitation frequency and pollination success is shown in Fig. 6 and Supplementary Fig. S6. The analysis of variable importance uncovered variations in trait preferences among different bee species (Fig. 6A). Particularly, nectar volume emerged as the most influential predictor of honey bee visitation, exerting a stronger effect on honey bees compared to bumble bees and other species. According to the partial dependence profile derived from random forest regression models, honey bee visitation increased with larger volumes of nectar secretion, reaching a plateau near 400 ml per 30 flowers (~ 13 ml/flower) (Supplementary Figure S6D). Additionally, honey bee visits were more strongly impacted by the diameter of the corolla aperture compared to other pollinator species, although this relationship followed a nonlinear pattern (Supplementary Figure S6D).

Nectar-foraging insects, encompassing honey bees, bumble bees, and other species, displayed marked associations with flower density (Fig. 6A). In contrast, pollen-foraging bumble bees displayed minimal responsiveness to flower density and were instead influenced by accessibility traits, namely corolla length and the corolla length-to-width ratio. Genotypes with protruded stigmas displayed higher visitation rates from honey bees and nectar-foraging bumble bees. However, this trait did not significantly contribute to the prediction of fruit set, fruit weight, or seed count (Fig. 6B).

## Discussion

Since the early stages of blueberry domestication, there has been extensive documentation of how pollination benefits fruit set and yield [26, 53–56]. Despite the routine use of pollination services, there is considerable variation in pollination success and subsequent yield reported among blueberry cultivars [4, 57, 58]. Flower morphological and nectar-related traits have shown to play significant roles in pollinator attraction, pollination efficacy, and fruit production [25–27]. Selective breeding of these traits has been proposed to address pollination deficits and improve yield outcomes [18, 59]. However, prior studies have been predominantly centered on northern highbush cultivars or a small selection of wild species and inter-specific hybrids [18, 25–27, 34]. Therefore, our understanding of genotypic diversity and the heritability of these traits in southern highbush blueberry remains limited, even though such knowledge is crucial for breeding efforts. In the present study, we assessed the diversity of flower morphology and nectar-related traits in 38 diverse southern highbush blueberry genotypes and evaluated their relative importance for pollinator attraction and pollination success within a breeding context.

The genotypes included in this study exhibited considerable diversity in all flower and nectar traits. Additionally, we found all traits to be moderately to highly heritable (0.30–0.77), which suggested that relevant genetic progress can be achieved for these traits throughout breeding cycles. This observation concurs with Lyrene (1994) [18], who reported that environmental conditions, including chilling units and temperature, had little impact on flower morphology, suggesting a strong genetic control of floral traits. Of the traits examined in this study, nectar volume exhibited the lowest heritability (h^2^ = 0.30). Nectar secretion is known to fluctuate with factors such as flower age, time of day, and cultivar, among others, which could affect the estimation of genetic parameters [24, 40, 60].

Floral traits were also able to predict pollinator visitation and seed count with moderate accuracy. Subsequent variable importance analysis revealed that honey bees displayed a clear preference for genotypes with larger nectar volumes, which is consistent with prior studies that demonstrated a positive correlation between nectar volume and honey bee visitation [35, 40, 61]. Our study also found a negative association between nectar volume and sugar concentration, indicating a dilution effect in nectar secretion. Distinct preferences were also apparent based on foraging tasks, with nectar-foraging bumble bees preferentially visiting genotypes with higher flower density and nectar volume. In contrast, pollen-foraging bumble bees showed a preference for flowers with short and wide corollas, facilitating stamen access. As the long tongue of bumble bees enables access to nectar from a broad range of flower shapes and sizes [62], preferences for a particular genotype may be primarily constrained by flower density and reward content rather than flower morphology.

Honey bees have previously been shown to prefer larger blueberry flowers with wide apertures [27]. To our surprise, we observed little to no relationship between flower size and honey bee visitation. Interestingly, random forest regression captured a nonlinear relationship between honey bee visitation and aperture diameter. Honey bee visits were highest for genotypes with small aperture diameters, narrower than 3 mm. However, honey bee visits were substantially lower for flowers with aperture diameters between 3 and 3.5 mm. Once aperture diameters exceeded a threshold of ~ 3.5 mm, honey bee visitation began to increase again. While it is still unclear why we observed this pattern, one possibility is that the narrow opening may restrict competing larger-bodied bees, such as carpenter bees and bumble bees, allowing for exclusive access to honey bees. Honey bees and bumble bees have been shown to compete for nectar, especially when hives are placed near bumble bee quads [63]. These competitive effects lead to altered foraging behavior and often decreased nectar availability [64]. In this case honey bees could be favoring flowers with smaller apertures that reduce competition from bumble bees.

A key expectation of this work was that genotypes exhibiting greater pollinator visitation frequency would also exhibit higher fruit set. However, we did not find this relationship to be statistically significant, possibly due to the constrained variability in fruit set observed. Although, other factors could have also limited our ability to discern this relationship, such as high rates of self-compatibility and parthenocarpy, which enables genotypes to set fruit with few or no pollinator visits [65, 66]. Environmental conditions during the bloom period, as well as plant health and fertility can further affect fruit set and fruit quality [67–69] pollinator dynamics, in which excessive visitation causes mechanical damage to flowers, or priority effects, where the identity of the initial floral visitor diminishes the success of subsequent visits [70, 71]. Still, our analyses showed that each genotype benefited in fruit set from increased pollinator visitation frequency. Moreover, the variable importance analysis revealed similar trends in the traits contributing to the predictive ability of both pollinator visitation and fruit set, suggesting an indirect relationship. Also contrary to our expectations, floral traits related to herkogamy, including anther-to-stigma distance, style length, and stigma protrusion, did not show strong effects on seed or fruit set.

Corolla length and the corolla length-to-width ratio were identified as the main drivers of pollen-foraging bumblebee visitation and all indices of pollination success, including fruit set, seed count, and fruit weight. This finding suggests a direct connection between flower morphology, pollinator recruitment, and fruit production. Previous research has demonstrated that pollen-foraging bumble bees are nearly five times more effective at pollen transfer than honey bees [16]. Therefore, it is possible that, despite the lower frequency of visits made by pollen-foraging bumble bees, they had a disproportionate effect on pollination indices due to their higher pollination efficacy. Furthermore, corolla length and the corolla length-to-width ratio had the highest heritability among all traits (h^2^ = 0.77), indicating that selective breeding for these traits may improve pollination outcomes at a higher rate than other flower characteristics.

The observed variation in the relative importance of blueberry floral traits across pollinator species and foraging behaviors is a novel finding with great implications for future efforts to enhance pollination. The high heritability and strong relationship of floral traits with pollinator visitation further emphasizes the potential of plant breeding to improve pollination outcomes and benefit growers through higher fruit production.

## Conclusions

This study delved into the multifaceted relationship between pollinators, flower morphology, pollination success, and yield parameters in blueberry, a subject that has been under-explored in the context of plant breeding. Our analyses confirmed the importance of nectar reward content and flower morphological traits in pollinator attraction. Moreover, we found these traits to be highly heritable, providing justification for breeding to improve pollination outcomes. Random forest and BayesB regression resulted in moderate to high predictive abilities for bee visitation and pollination success using floral traits. Subsequent variable important analysis revealed distinct preferences between honey bees and bumble bees conducting different foraging tasks. Nectar volume was found to be the most important trait mediating honey bee visitation frequency, while bumble bees were instead influenced by flower accessibility traits, including corolla length and the length-to-width ratio. Flower density had a substantial impact on all pollinator species, with a pronounced effect on nectar-foraging bumble bees and non-honey bee species. These findings emphasize the critical role of floral density and flower accessibility in the pollination landscape. While the relationship between flower visitation frequency and fruit set was not significant in our analyses, we expect that using more pollinator-attractive cultivars on farms experiencing pollination deficits may result in higher production, given the generally positive effect of pollination on yield. Additionally, the high heritability for floral traits reported here can provide valuable insight for the prioritization of traits for the genetic improvement of pollinator attraction, and ultimately yield in an outcrossing and pollinator-dependent crop such as blueberry.

### Electronic supplementary material

Below is the link to the electronic supplementary material.

**Supplementary Material 1: Fig. S1** Histogram distribution of flower morphological traits in both years. CL, corolla length (cm); CW, corolla width (cm); LW, ratio of corolla length-to-width; SZ, flower size (cm^3^); AP, aperture diameter (cm); SL, style length (cm); SP, stigma protrusion from corolla (cm); ASD, anther-to-stigma distance (cm); FOB, flowers on bush (flowering density); NV, nectar volume (µL); NSC, nectar sugar content (°BRIX); FS, fruit set; SC, seed count of 10 berries; FW, fruit weight of 25 berries (g)

**Supplementary Material 2: Fig. S2** Pollinator visits observed per minute for each genotype between years. Colors indicate pollinator species and foraging behavior. Other species included carpenter bees (*Xylocopa virginica* and *X. micans*), flower wasps (*Scoliid* spp.), hover flies (Syrphidae), and the southeastern blueberry bee (*H. laboriosa*)

**Supplementary Material 3: Fig. S3** The temperature (°C) (green line) and cloud-cover percent during the 2021 and 2022 growing seasons. Julian date is presented as day of the year

**Supplementary Material 4: Fig. S4** The spatial distribution of fruit set (FS), honeybee visitation, bumblebee visitation, and other flower visitors across rows and columns (referring to the positions of each genotype in the field) for the 2021 and 2022 seasons

**Supplementary Material 5: Fig. S5** Outliers removed due to high number of seeds with low pollinator observations (A) and high rates of self-compatibility, measured as ripe fruit set in response to ten self-pollinated flowers (B), which could inflate fruit set at lower visitation rates

**Supplementary Material 6: Fig S6** The partial dependence profile for the relationship between pollination indices (A-C) and pollinator visitation frequency (D-G) with each individual flower trait based on BayesB and Random Forest regression, respectively. AP, aperture diameter; ASD, anther-to-stigma distance; CL, corolla length; CW, corolla width; FOB, flowers on bush (flowering density); LW, ratio of corolla length-to-width; NSC, nectar sugar content; NV, nectar volume; SL, style length; SP, stigma protrusion from corolla; SZ, flower size

## Data Availability

The datasets used in this study are available from the corresponding author upon reasonable request.

## Abbreviations

AP: Aperture diameter
ASD: Anther-to-stigma distance
BLUP: Best-linear unbiased prediction
CL: Corolla length
CW: Corolla width
FOB: Flowers on bush
FS: Fruit set
FW: Fruit weight of 25 berries
LW: Ratio of corolla length-to-width
NSC: Nectar sugar content
NV: Nectar volume
PLS: Partial least squares
REML: Restricted maximum likelihood
RF: Random forest
RMSE: Root-mean square error
RR: Ridge-regression
SC: Seed count of 10 berries
SL: Style length
SP: Stigma protrusion from corolla
SZ: Flower size
