# Supplementary figures and images for: Genotypic variation in blueberry flower morphology and nectar reward content affects pollinator attraction in a diverse breeding population
Source: BMC Plant Biol. 2024 Aug 29;24:814. doi: 10.1186/s12870-024-05495-6 (PMC11360736; doi:10.1186/s12870-024-05495-6)

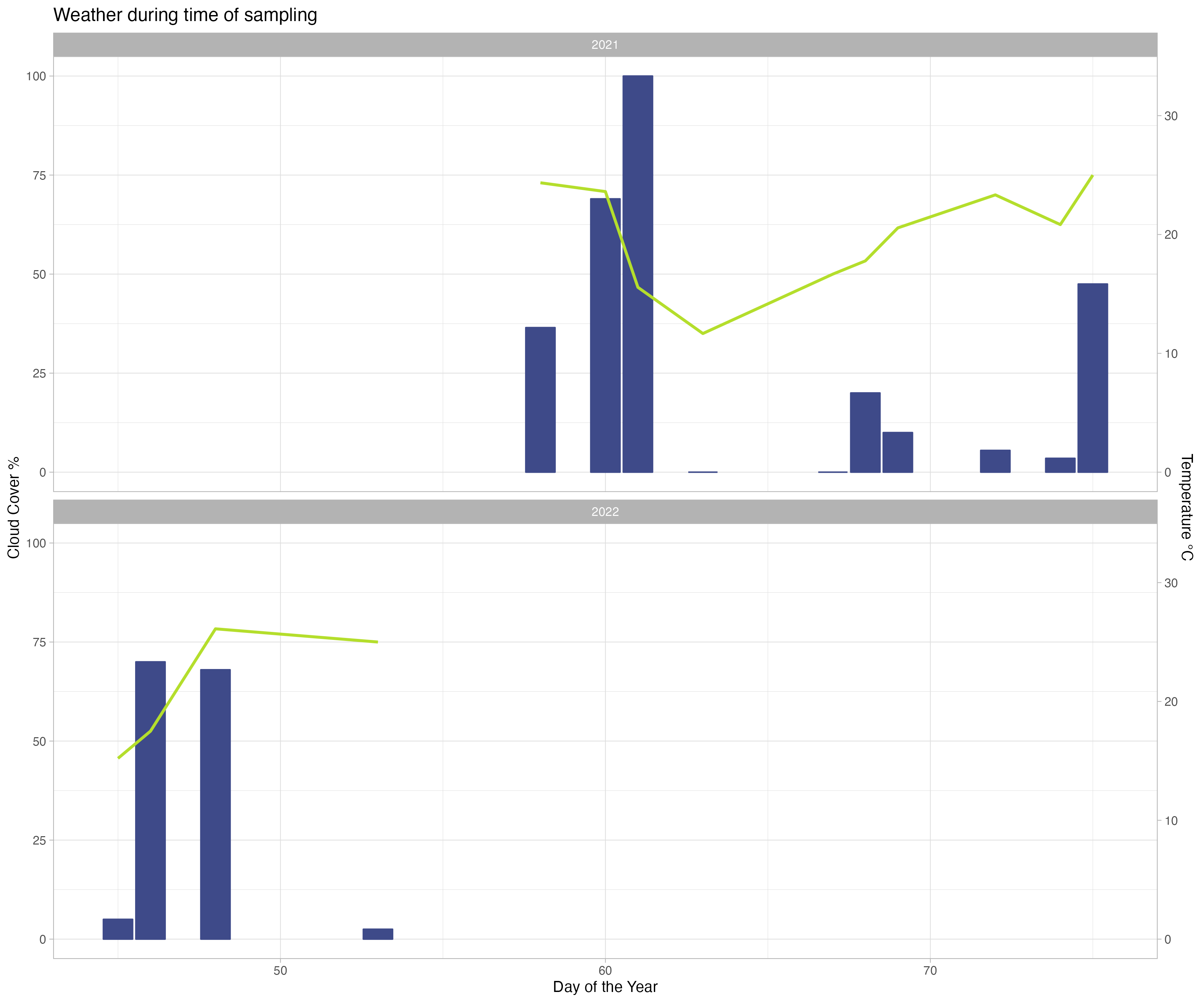

Supplement: Supplementary file 1 — Supplementary Material 1: Fig. S1 Histogram distribution of flower morphological traits in both years. CL, corolla length (cm); CW, corolla width (cm); LW, ratio of corolla length-to-width; SZ, flower size (cm3); AP, aperture diameter (cm); SL, style length (cm); SP, stigma protrusion from corolla (cm); ASD, anther-to-stigma distance (cm); FOB, flowers on bush (flowering density); NV, nectar volume (µL); NSC, nectar sugar content (°BRIX); FS, fruit set; SC, seed count of 10 berries; FW, fruit weight of 25 berries (g) [file 12870_2024_5495_MOESM1_ESM.png]

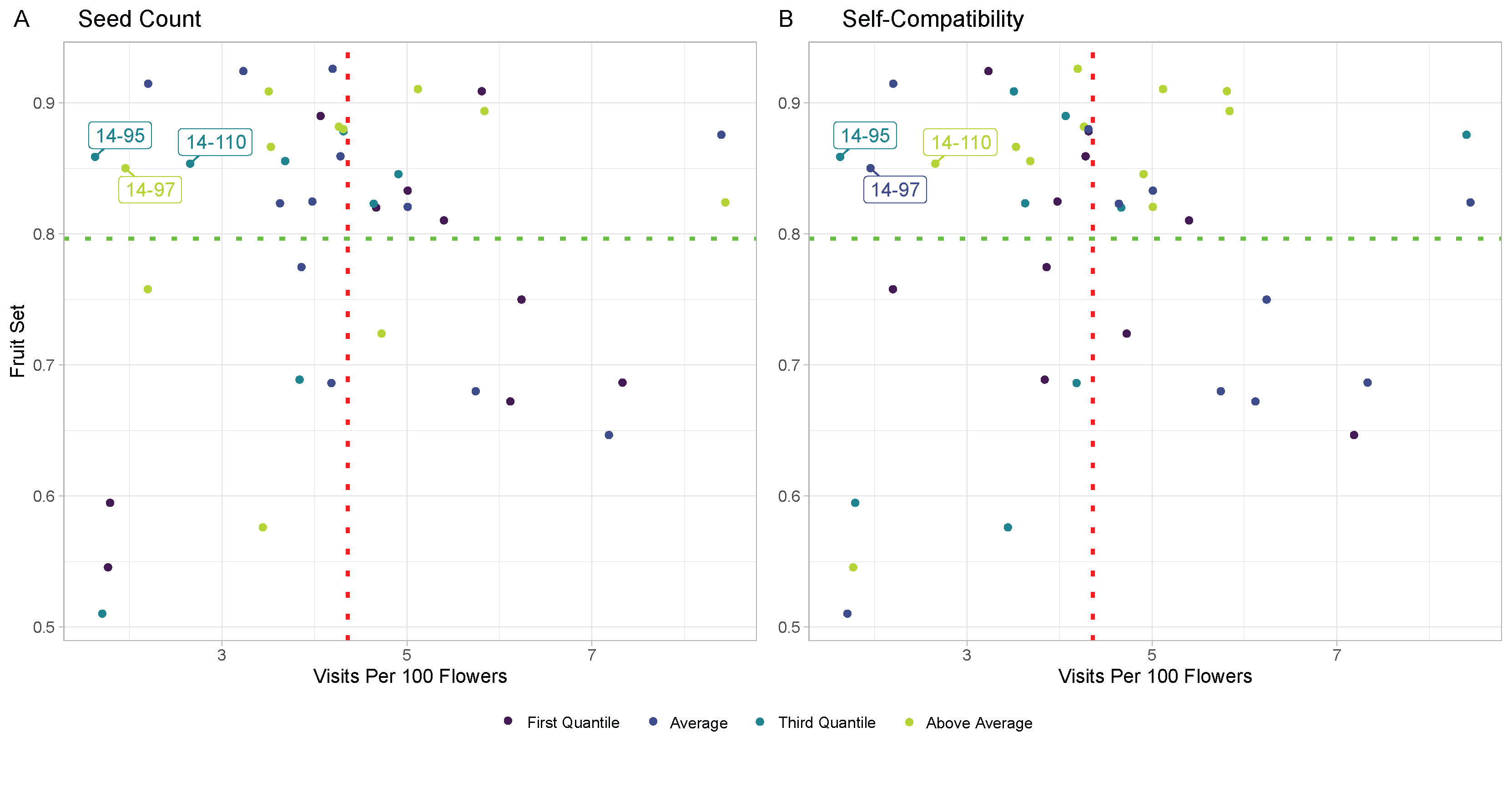

Supplement: Supplementary file 2 — Supplementary Material 2: Fig. S2 Pollinator visits observed per minute for each genotype between years. Colors indicate pollinator species and foraging behavior. Other species included carpenter bees (Xylocopa virginica and X. micans), flower wasps (Scoliid spp.), hover flies (Syrphidae), and the southeastern blueberry bee (H. laboriosa) [file 12870_2024_5495_MOESM2_ESM.png]

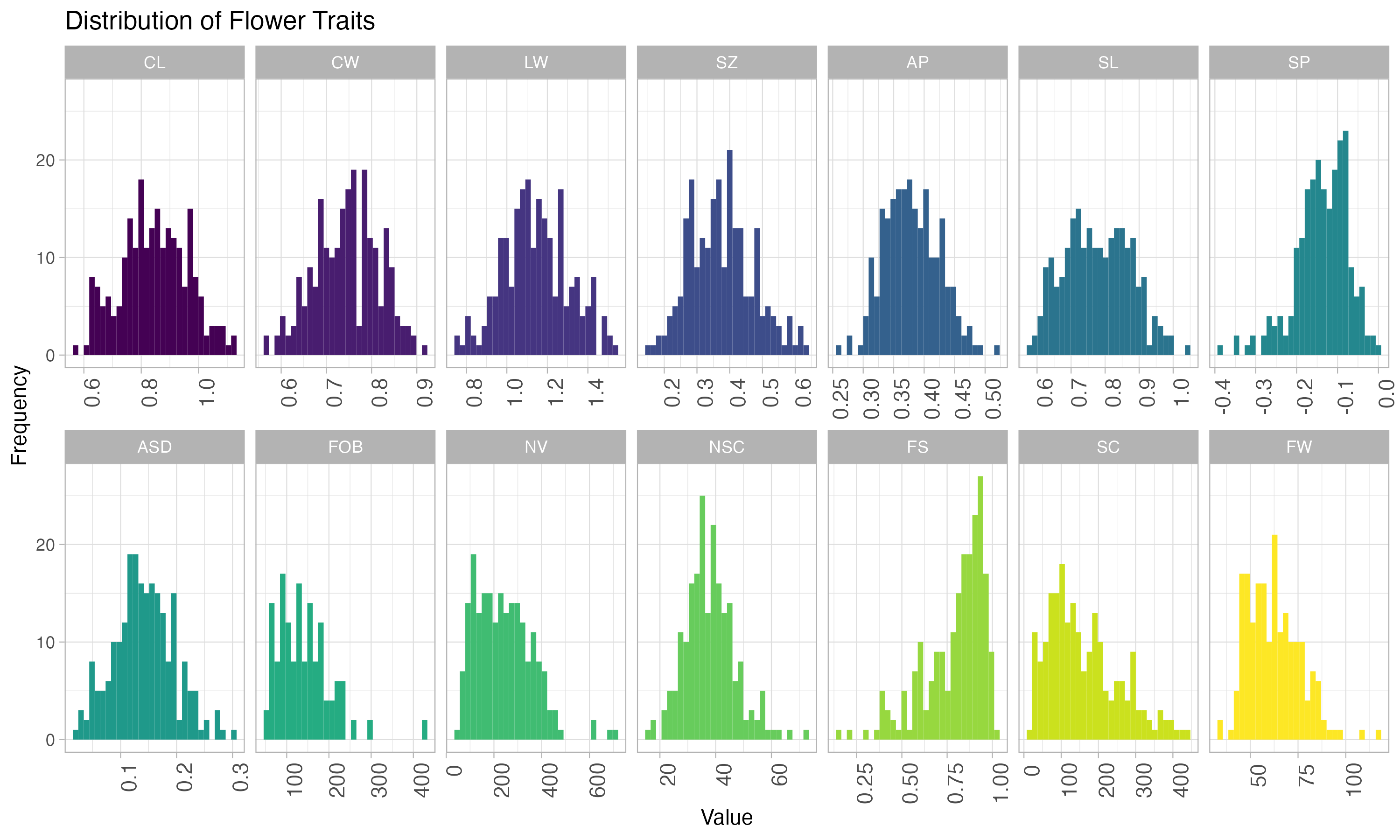

Supplement: Supplementary file 3 — Supplementary Material 3: Fig. S3 The temperature (°C) (green line) and cloud-cover percent during the 2021 and 2022 growing seasons. Julian date is presented as day of the year [file 12870_2024_5495_MOESM3_ESM.tiff]

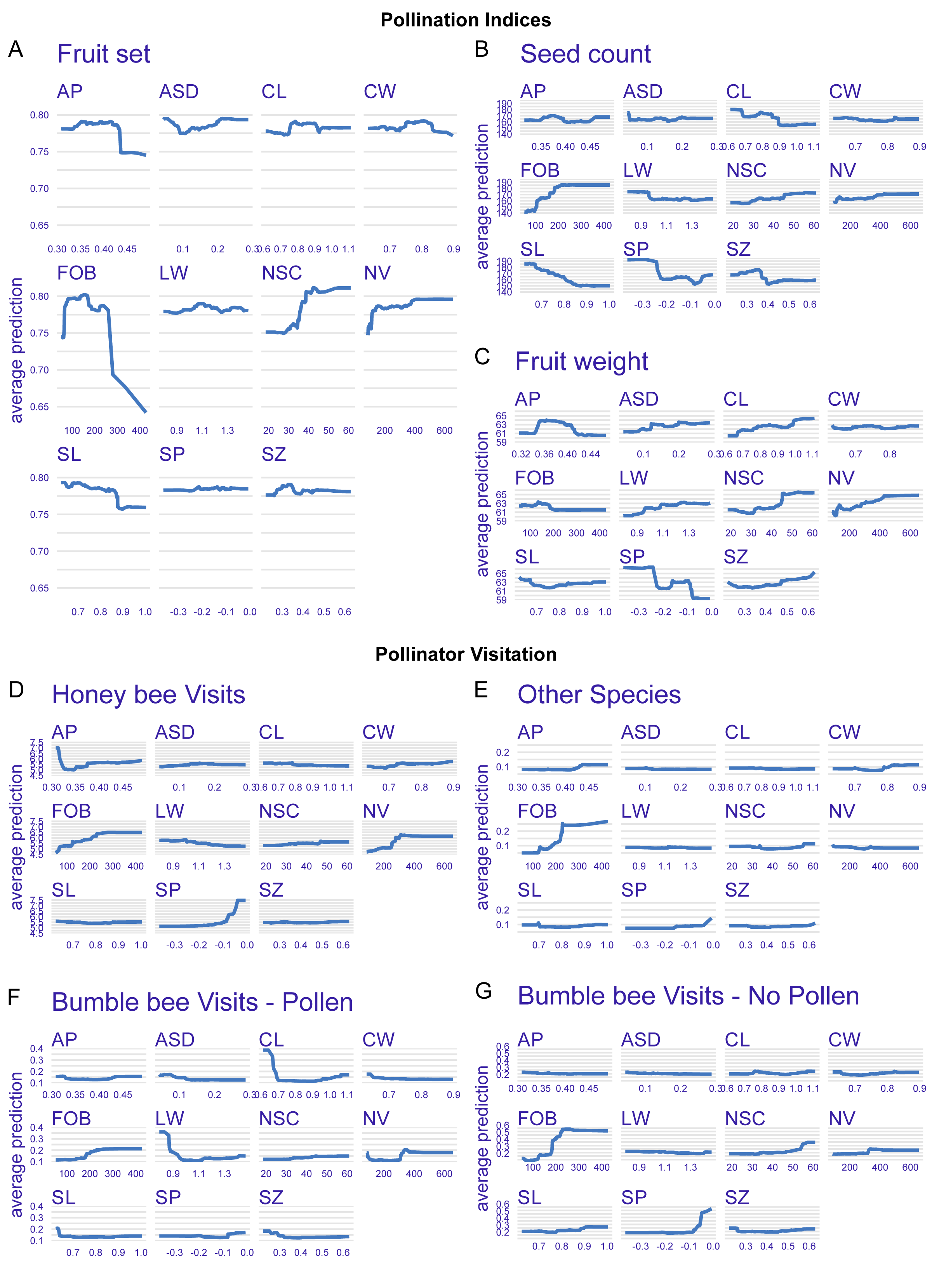

Supplement: Supplementary file 4 — Supplementary Material 4: Fig. S4 The spatial distribution of fruit set (FS), honeybee visitation, bumblebee visitation, and other flower visitors across rows and columns (referring to the positions of each genotype in the field) for the 2021 and 2022 seasons [file 12870_2024_5495_MOESM4_ESM.tif]

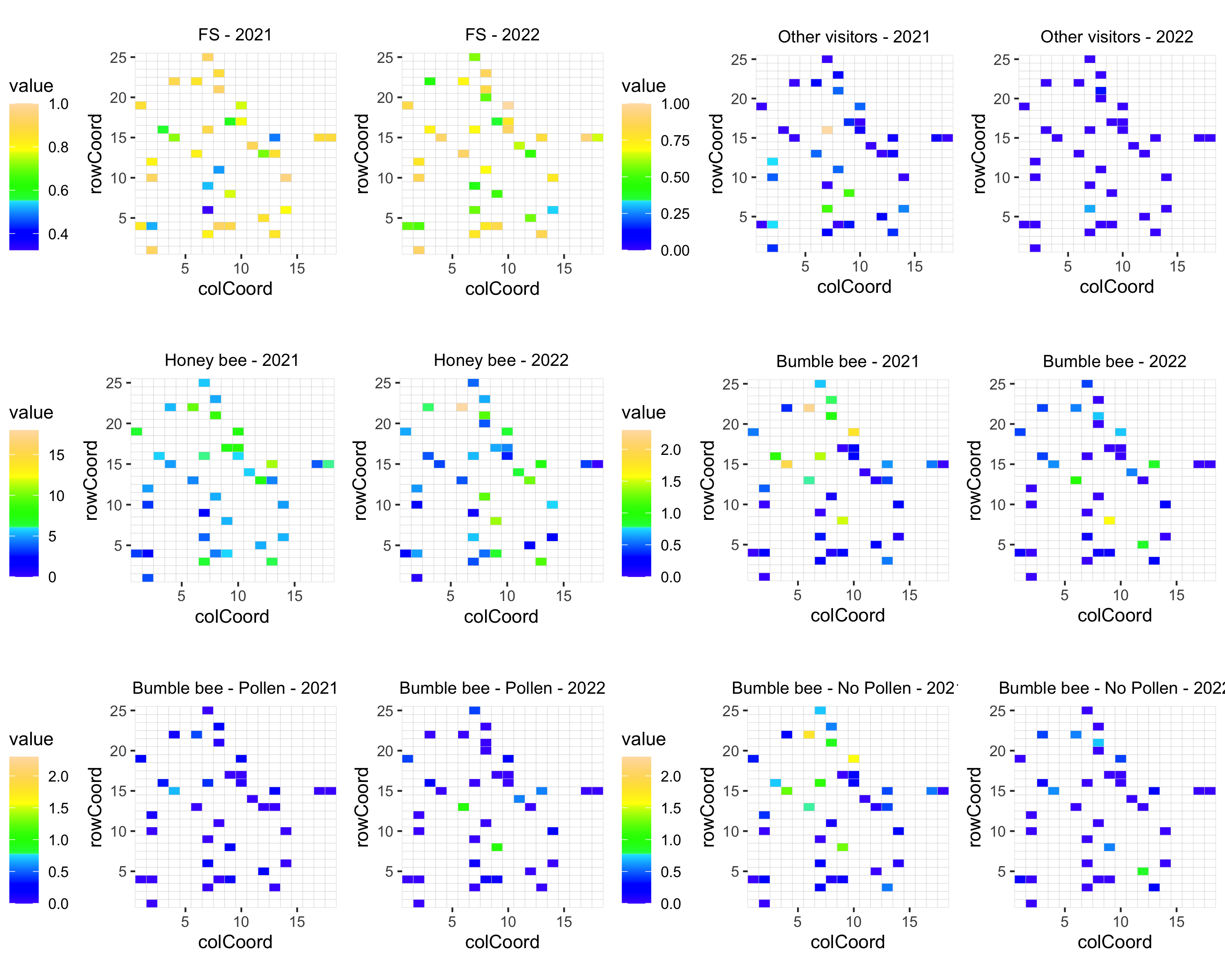

Supplement: Supplementary file 5 — Supplementary Material 5: Fig. S5 Outliers removed due to high number of seeds with low pollinator observations (A) and high rates of self-compatibility, measured as ripe fruit set in response to ten self-pollinated flowers (B), which could inflate fruit set at lower visitation rates [file 12870_2024_5495_MOESM5_ESM.tiff]

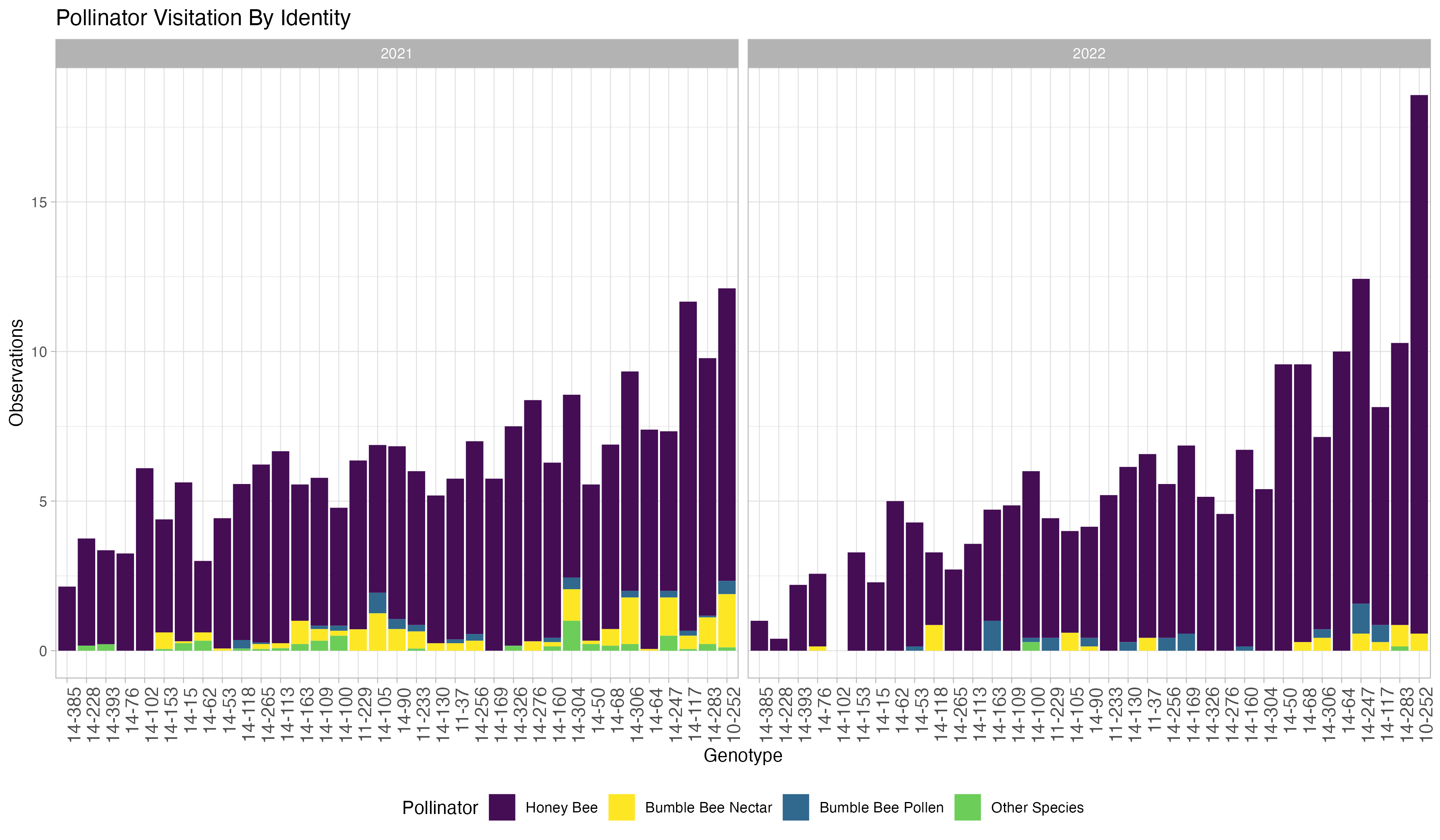

Supplement: Supplementary file 6 — Supplementary Material 6: Fig S6 The partial dependence profile for the relationship between pollination indices (A-C) and pollinator visitation frequency (D-G) with each individual flower trait based on BayesB and Random Forest regression, respectively. AP, aperture diameter; ASD, anther-to-stigma distance; CL, corolla length; CW, corolla width; FOB, flowers on bush (flowering density); LW, ratio of corolla length-to-width; NSC, nectar sugar content; NV, nectar volume; SL, style length; SP, stigma protrusion from corolla; SZ, flower size [file 12870_2024_5495_MOESM6_ESM.png]
